# What is the relationship between exposure to environmental pollutants and severe mental disorders? A systematic review on shared biological pathways

**DOI:** 10.1016/j.bbih.2024.100922

**Published:** 2024-12-12

**Authors:** Pierluigi Catapano, Mario Luciano, Salvatore Cipolla, Daniela D'Amico, Alessandra Cirino, Maria Chiara Della Corte, Gaia Sampogna, Andrea Fiorillo

**Affiliations:** Department of Psychiatry, University of Campania “L. Vanvitelli”, 80138, Naples, Italy

**Keywords:** Climate change, Pollutants, Depression, Schizophrenia, Inflammation, Oxidative stress, **Secondary keywords**, Environment, Biological mechanisms

## Abstract

Severe mental disorders are multi-dimensional constructs, resulting from the interaction of genetic, biological, psychosocial, and environmental factors. Among the latter, pollution and climate change are frequently being considered in the etiopathogenesis of severe mental disorders. This systematic review aims to investigate the biological mechanisms behind the relationship between environmental pollutants, climate change, and mental disorders. An extensive literature search was performed on PubMed, Scopus, and APA PsycInfo databases according to the PRISMA guidelines. Articles were considered eligible if they involved humans or animals examining the association between exposure to environmental pollutants and if the resulting biological mechanisms that may have an impact on mental health and may support or even cause severe mental disorders (SMD) are assessed. For this reason, only studies dealing with biomarkers or biological pathways were taken into account. The 47 papers included in the review were divided into two groups: those conducted on human participants (15 studies) and those utilizing animal models (31 studies); one study included both humans and animals. Studies carried out with humans, which are mainly focused on measuring the impact of particulate matter (PM_2.5_ and PM_10_) exposure on mental health, showed an increased risk of depression or psychotic relapses through the inflammation and oxidative stress pathways, or through the alteration of the hypothalamic-pituitary-adrenal (HPA) axis. Animal models showed the potential impact of pollution on brain functioning through increased inflammatory responses, oxidative stress, HPA axis disruption, hippocampal damage, and neurotransmitters dysregulation. Our findings show that environmental pollutants have an impact on human mental health through different biological pathways. The biological mechanisms by which environmental pollution and climate change influence the onset and exacerbation of severe mental disorders are complex and include gene expression, inflammation, oxidative stress, and anatomical brain changes. A better understanding of those pathways is important for the progress of knowledge on the pathophysiology of severe mental disorders according to the one health model, that promotes a collaborative, multisectoral, and transdisciplinary approach across various levels to optimize health outcomes by recognizing the interconnectedness of humans, animals, plants, and their shared environment.

## Highlights

• Mechanisms linking exposure to pollutants and mental disorders have been reviewed.
• PM exposure linked to severe mental disorders via different biological pathways.
• Animal models show pollution impacts brain function and neurotransmitter regulation.
• Insights into biological pathways connecting environmental factors and mental health.
• "One Health" approach in understanding mental health and environmental impact.

## 1 Introduction

In recent years, researchers have increasingly focused on the 'One Health' concept, that highlights the interconnectedness of human, animal, and environmental health. By examining the environmental impact on severe mental disorders (SMD), the present systematic review underscores the need to understand the complex biological, ecological, and social interactions that influence mental health outcomes (CDC About One Health Available). The onset of mental disorders is the result of a complex interaction between genetic, biological, psychological, social, and environmental factors (Stein et al., 2022; Roberts et al., 2023). Thus, the pathophysiological bases of mental illnesses are multidetermined, and their understanding may significantly improve mental health care, in terms of prevention and treatment strategies (Dragioti et al., 2023).

Similarly to cancer and other complex disorders, mental illnesses are often the result of the interaction between genetic predisposition and environmental factors (Fiorillo and Giordano, 2022). Among the environmental factors, climate change and pollution have been recently considered among possible contributors to the onset of a variety of mental disorders, also following the Lancet Countdown on Climate Change and Health (Watts et al., 2018).

Climate change and environmental pollution are interdependent, each amplifying the other. Rising temperatures increase pollutants like ozone (O_3)_ and particulate matter by enhancing chemical reactions and altering atmospheric conditions (Reinmuth-Selzle et al., 2017). For example, higher temperatures boost ground-level O_3_ formation through accelerated photochemical reactions from vehicle and industrial emissions (Cabral et al., 2019). Conversely, pollution contributes to climate change by releasing greenhouse gases and aerosols, such as black carbon, which absorb sunlight and trap heat, intensifying the greenhouse effect (De Sario et al., 2013). Therefore, even when aiming to focus on the effects of environmental pollutant exposure on human health, it is essential to preface this discussion with the pressing issue of climate change, as pollution is an integral component of this global challenge.

With respect to air pollution, ultrafine particles (UFPs; diameter ≤0.1 μm), fine particles (PM_2.5_; diameter ≤2.5 μm) (commonly found in urban environments and originating from vehicle emissions, industrial processes, and combustion of fossil fuels) have a negative impact on brain health (Schraufnagel, 2020; Salvi and Salim, 2019). It is also reported that O_3_ also impacts brain health metrics, as evidenced by numerous studies that demonstrate its neuroinflammatory effects across various age groups (Morrel et al., 2024; Cotter et al., 2024; Tyler et al., 2018). Similarly, nitrogen dioxide (NO_2_) another harmful pollutant primarily produced from road traffic and fossil fuel combustion, contributes to respiratory and neurological issues (Weichenthal et al., 2020). Heavy metals (often found in contaminated water, soil, and air, typically resulting from industrial discharges) and pesticides (used extensively in agriculture to control pests, and released into the environment or absorbed into the body through ingestion of contaminated food) also have cognitive and neurological outcomes, which might be persistent after exposure to neurotoxic substances (Reuben et al., 2022), especially in urban cities.

In urban areas, city traffic represents one of the main sources of atmospheric pollution. Since the proportion of the world's population living in urban areas will be 68% by 2050, and given the high impact of mental health on the social and economic growth of the world population (The Lancet Global Health Mental Health Matters, 2020), it is imperative to increase the understanding of mechanisms through which air pollution affect mental health.

When considering the broader context, it is important to note that environmental pollutants, such as fine particulate matter and heavy metals, have been shown to disrupt immune processes, which can, in turn, impact mental health. For example, research has highlighted the role of these pollutants in altering gut microbiome composition, which is increasingly recognized as a critical factor in brain health and immune function (Singh et al., 2022)**.**

Several studies have tried to understand the biological link between air pollution and mental health (Borroni et al., 2022a; Liu et al., 2020). Although the biological mechanisms by which pollutants may lead to a biological dysfunction in the brain circuits are still poorly investigated, the inflammatory response triggered by pollutants can exacerbate stress reactions, further linking environmental factors to mental health outcomes (Hahad et al., 2020). In particular, air pollution may affect several biological pathways, including oxidative stress and inflammation. Previous research stated that oxidative stress may reduce dopamine production (Nieoullon, 2002), while neuroinflammation can impact on astrocytes and microglia activity, contributing to sustained brain inflammation (Yatham, 2023a). Moreover, increased levels of pro-inflammatory cytokines might excessively activate the kynurenine pathway (KP), resulting in decreased tryptophan and serotonin levels, potentially leading to the onset of depressive symptoms (Zádor et al., 2021). In addition, pro-inflammatory mediators have an important role in the attenuation of the regulatory glucocorticoid-mediated feedback, involved in response to stress and implicated in depression and cancer (Polityńska et al., 2022).

Other mechanisms through which the environment can influence the onset or worsening of severe mental disorders have been reported with reference to the pathogenesis of schizophrenia (Mesholam‐Gately et al., 2023). In fact, people with schizophrenia have higher levels of cytokines in peripheral blood, along with responsive microglia in the central nervous system (Khandaker et al., 2015). Microglial reaction may lead to abnormal synaptic pruning, loss of cortical gray matter, and damage to critical brain areas, such as the prefrontal cortex and the hippocampus, leading to a worsening of psychotic symptoms (Colizzi et al., 2023).

Based on these premises, the present systematic review aims to assess the current level of knowledge on biological patterns by which pollutants can affect mental disorders, both in human and animal models. It has to be noted that although animal models can exhibit symptoms analogous to human mental disorders, they cannot be formally diagnosed in the same way as humans. Nonetheless, these models serve as a valuable foundation for investigating the underlying biological mechanisms linking pollution and mental health.

## 2 Methods

### 2.1 Search strategy

An extensive literature search for relevant articles has been performed from inception up to September 10, 2024 on PubMed, Scopus and APA PsycInfo, by entering the following search keys: ((("Environmental Pollutants"[Mesh]) OR "Climate Change"[Mesh]) AND (((("Schizophrenia Spectrum and Other Psychotic Disorders"[Mesh]) OR "Bipolar Disorder"[Mesh]) OR "Depression"[Mesh]) OR "Depressive Disorder"[Mesh])) AND ((((("Inflammation"[Mesh]) OR "Biological Factors"[Mesh]) OR "Biomarkers"[Mesh]) OR "Hormones"[Mesh]) OR "Metabolism"[Mesh]), on PubMed; TITLE-ABS-KEY (((environmental AND pollutants) OR (environmental AND pollutant) OR (air AND pollutant) OR (air AND pollutants) OR (water AND pollutants) OR (water AND pollutant) OR (climate AND change)) AND ((schizophrenia) OR (psychosis) OR (psychotic AND disorders) OR (bipolar AND disorder) OR (depression) OR (depressive AND disorder)) AND ((inflammation) OR (biological AND factors) OR (biomarkers) OR (hormones) OR (metabolism))) AND (LIMIT-TO (LANGUAGE, "English")), on Scopus; ("Environmental Pollutants" OR "Environmental Pollutant" OR "Air pollutant" OR "Air pollutants" OR "Water pollutants" OR "Water pollutant" OR "Climate Change") AND ("Schizophrenia" OR "Psychosis" OR "Psychotic Disorders" OR "Bipolar Disorder" OR "Depression" OR "Depressive Disorder") AND ("Inflammation" OR "Biological Factors" OR "Biomarkers" OR "Hormones" OR "Metabolism"), on APA PsycInfo. We included climate change as a broad search term to capture studies on pollutants that address this latter topic as a secondary focus, since the two issues are interrelated. The search method has been carried out according to the Preferred Reporting Items for Systematic Review and Meta-Analysis (PRISMA) statement, as applicable (Page et al., 2021). No review protocol was prepared.

Inclusion criteria have been established to determine eligibility, as follows: a) studies involving animals or humans exposed to environmental pollutants, with no restrictions on patients’ gender, ethnicity, or age; b) studies analyzing biological mechanisms underlying the relationship between exposure to environmental pollutants or climate change and mental health problems; c) articles reporting psychiatric measures of outcome, such as behavioral tests for animals and validated psychometric tests or hospitalization/relapsing rate or suicide attempts for humans.

The following article types were excluded from the analysis: a) systematic reviews, meta-analyses, preliminary studies, editorials, book chapters, letters and notes; b) articles written in a language other than English whose data could not be obtained from other records.

Reference lists of included articles were screened to identify additional relevant studies.

### 2.2 Selection process

**Fig. 1:**
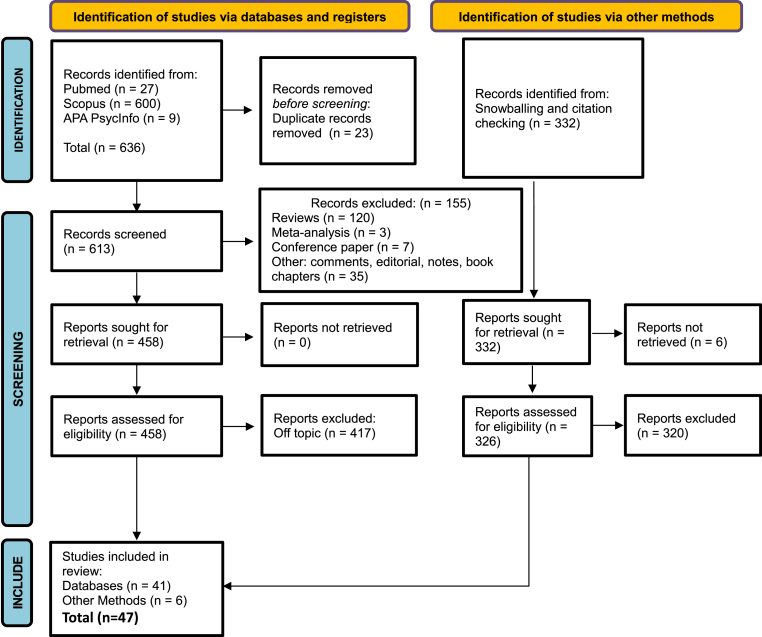
PRISMA Flowchart of the included studies.

A total of 636 papers were identified. After controlling for duplicates, 23 papers were removed. Of the remaining papers, 155 were excluded since they were systematic or narrative reviews (120), meta-analyses (3), conference papers (7), editorials (10), notes (2), comments (1), letters (3), and book chapters (9). After a full-text reading, a total of 417 articles were removed as they did not meet the inclusion criteria. Six more papers were added by snowballing and citation checking. Finally, 47 papers were included in the review analysis. The selection process is shown in Fig. 1.

Four reviewers independently evaluated the studies retrieved from database searches through three phases: literature search, title and abstract screening, and full-text screening. A senior researcher was consulted as necessary. Three researchers extracted relevant data, which was then triple checked for accuracy by two senior researchers. Any disagreements among reviewers were resolved through discussion, aided by a senior researcher. Inter-rater reliability, referring to the level of agreement between researchers, has been calculated, with a Cohen's kappa score of 0.85, which indicates nearly perfect consensus among researchers (McHugh, 2012).

### 2.3 Risk of bias assessment

Two researchers independently assessed each selected observational study for the risk of bias (RoB) using the ROBINS-E tool (Higgins et al.), a structured instrument widely used for evaluating RoB in this type of studies. The overall risk of bias was rated from “Low risk of bias, except for concerns about uncontrolled confounding” to “High” for the observational studies. Referring to experimental studies, the RoB was evaluated via RoB II, based on criteria recommended in the Cochrane Handbook for Systematic Reviews of Interventions (Cochrane). The overall Rob in experimental studies was moderate. Supplementary Tables S1 and S2 detail the considered domains and subdomains. Disagreements were resolved through discussion among the two authors or by consulting a third author.

## 3 Results

The 47 papers included in the review were grouped in two categories: 1) studies involving animal models (31 papers); and 2) studies involving humans (15 papers). One study involved both human and animal models and was considered separately. Below, we present the principal findings of our literature analysis, systematically organized according to the pollutants studied. The implications of these findings will be addressed in the subsequent discussion section.

### 3.1 Studies involving humans

Of the 15 studies carried out on humans, 6 adopted a cohort design, 5 were cross-sectional and 4 were case-control studies. None of them was a randomized control trial (RCTs). Studies included in this group are reported in Table 1. The sample sizes of studies ranged from 24 (Wei et al., 2022) to 7794 (Xu et al., 2024) participants, with a total sample of 14150, of which 5057 (35.7%) were male and 9093 (64.3%) female.

**Table 1:** Studies involving humans.

Author (year); *Type of study* (Country)	Population	SMD	Pollutant	Main findings
		Psychiatric assessment	Mechanism	
Xu et al. (2024) *Cohort study* (China)	7794 individuals	Depression	PM_2.5_	A positive relationship between chronic exposure to ambient PM_2.5_and depressive symptoms was found. The association between PM_2.5_and depressive symptoms appears to be partially mediated through the presence of metabolic risk factors (hypertension, dyslipidemia, diabetes).
		PHQ-9	Metabolic risk factors are the pathway between long-term exposure to PM_2.5_ and depression.	
Abo-el-Ata et al. (2023) *Cross-sectional study* (Egypt)	180 individuals	Depression; Neuropsychological functions	PM_10_; CO; CO_2_; SO_2_; NO_2_; VOCs	Occupational exposure to air pollutants among pottery workers is associated with impairment in neuropsychological functions and increased risk of depression, with a corresponding increase in the serum level of 4 HNE, which is a biomarker for oxidative stress.
		- HAM-D; - Digit symbol substitution test; - Benton visual retention test; - Digit span; - Fatigue severity scale; - Neurobehavioral validated and reliable test batteries; - Epworth sleep scale	Oxidative stress	
Bjørklund and Semenova, 2023 *Cross-sectional study* (Kazakhstan)	1881 individuals	Depression	Pollution resulting from industrial or military activity.	The exposed residents had higher median serum concentrations of cortisol (except for group 1), lowest median serum levels of ACTH and highest prevalence of primary hypoadrenalism. The median total PHQ-9 scores were the lowest in residents of control sites. Both serum cortisol and ACTH correlate with moderate, moderately severe, and severe depression. Therefore, the findings highlight the potential impact of environmental pollution on stress hormone levels and depression.
		PHQ-9	Hypothalamic-pituitary-adrenal (HPA) axis.	
Lei et al. (2023) *Historical cohort study* (China)	332 patients with schizophrenia	Schizophrenia	PM_2.5_	The recurrence of schizophrenia was influenced by the combined effects of PM_2.5_ concentrations, the YWHAB gene polymorphism locus rs6031849, and gender (with females being more at risk than males). Prolonged exposure to elevated PM_2.5_ levels had the most significant impact on relapse.
		Relapse rate	Interaction with YWHAB gene polymorphism (locus rs6031849)	
Yuan et al. (2023) *Observational prospective study* (China)	40 students from Medical University	Anxiety, Depression	PM_2.5_	Acute exposure to PM_2.5_ and its depositions may elevate health risks by raising blood pressure, inducing anxiety and depression, and altering the urinary metabolomic profile through activation of the cAMP signaling pathway.
		SAS, SDS	cAMP signaling pathway activation	
Bao et al. (2022) *Cohort study* (China)	989 individuals	Depression	Phthalates	Elevated concentrations of PAEs, specifically MEHP, MBzP, and MBP, were linked to a higher likelihood of depressive symptoms among older adults. Chronic inflammation emerged as a significant risk factor for these symptoms. Models examining mediating effects indicated that IL-6 and generalized inflammation factors (a composite measure that includes various systemic inflammatory biomarkers, such as interleukines and CRP, which collectively reflect the body's inflammatory response) played a partial mediating role in the relationship between MEHP exposure and the heightened risk of depressive symptoms.
		GDS-30	Neuroinflammation	
Wei et al. (2022) *Clinical trial* (China)	24 patients with schizophrenia	Schizophrenia	PM_2.5_; PM_10_	PM exposure may increase the risk of relapse of schizophrenia and decrease the function of the antioxidant system, especially the level of T-AOC. Moreover, a decrease in T-AOC might have the potential mediation effect on the association between PM_2.5_ exposure and the risk of schizophrenia relapse.
		ESS	Oxidative stress	
Ahlers and Weiss, 2021 *Cohort study* (USA)	179 individuals	Depressive symptoms	PM_2.5_	Findings suggest pregnancy may be a critical window of sensitivity to PM_2.5_ exposure that escalates depression risk and induces activation of the HPA axis, evidenced in greater overall cortisol concentration.
		PHQ-9	DNA methylation and increased oxidative stress, neuroinflammation, cerebrovascular damage, neurodegeneration, and dysregulation of the HPA axis.	
Gao et al. (2021) *Prospective cohort study* (China)	58 patients with schizophrenia in remission phase	Schizophrenia	PM_2.5_	Elevated PM_2.5_ increases the risk of relapse of schizophrenia, and the harm has a short-term hysteresis effect. PM_2.5_increases the risk of overall relapse primarily by aggravating non-psychotic symptoms of schizophrenia. The imbalance of inflammatory cytokine IL-17 may play an intermediary role in the process of PM_2.5_ increasing the risk of relapse.
		ESS	Neuroinflammation	
Gaum et al. (2019) *Observational longitudinal study* (Germany)	116 individuals	Depression	Polychlorinated and Hydroxylated Biphenyls (PCBs) and their hydroxylated metabolites OH-PCBs	This study gives first hints for an underlying pathomechanism that may explain the association of occupational and inhalative PCB exposure and depressive symptoms. The findings show that a physiological process involving the DA and thyroid system may be responsible for depressive symptoms after PCB exposure. Nevertheless, more research is necessary to support these findings.
		PHQ-9	PCBs can displace T4 by binding to TTR itself, being transported into the brain and disturbing DA-synthesis, Consequently, (fT4) increases when PCBs bind to TTR.	
Jia et al. (2018) *Experimental study* (China)	Human model: 12 non-smoking male adults. Mice model: 20 C57 BL/6 J male mice	Depressive behaviors	PM_2.5_	By the animal models, a causal relationship between PM exposure and mental disorder was found. Heavy particulate pollution has the possibility to aggravate the symptoms of mental illness, and the risks of mood-related behaviors disorder in human.
		Humans: no test Mices: OFT, L/DBT, EPM test, FST, and SIT	Neurotoxicity, and impaired the neuron structure in mice hippocampus	
Gaum et al. (2017) *Observational longitudinal study* (Germany)	178 individuals	Depression	PCBs: LPCBs, HPCBs dlPCBs	High PCB exposure is associated with more depressive symptoms and the relationship between different types of PCB exposure and depressive symptoms may be mediated by alterations in dopamine metabolism over time.
		BDI-II	PCBs exposure is associated with lower concentrations of urinary HVA, the main dopamine metabolite.	
Ng et al. (2015) *Prospective cohort study* (Taiwan)	166 mother-infant pairs	Behavioral problems in children including symptoms of anxiety and depression	Mercury	Carriers of the APOE e4 allele and elevated cord blood mercury (Hg) concentrations showed increased scores in all Child Behavior Checklist (CBCL) syndromes compared to the reference group. This suggests that elevated Hg in cord blood likely enhances the risk of deficit behavior in preschool children who are APOE e4 carriers. The findings also supported the idea that APOE e4 may modify the relationship between Hg and child neurodevelopment.
		Child Behavior Checklist 1.5/5 (CBCL/1.5–5)	MethylMercury neurotoxicity - modifying effects of APO E genetic variants on MeHg neurotoxicity.	
Mukherjee et al. (2014) *Cross-sectional study* (India)	654 people enrolled from Arsenic endemic area (groundwater As 11–50 mg/L; n = 342) and control areas (As level 10 mg/L; n = 312) Gender: F	Neurobehavioral symptoms, Depression	Arsenic (As); PM_2.5_; PM_10_	The percentage of P-selectin-expressing platelets significantly correlated with BDI, and platelet serotonin showed a strong negative correlation with BDI score, indicating the possible involvement of platelet activation and secretion of contents of alpha granules (such as P-selectin) and dense granules (such as serotonin) in the pathophysiology of depression among As-exposed women. Chronic low-level As exposure (11–50 μg/L), even after adjusting for potential confounders, was a significant positive determinant for neurobehavioral symptom prevalence and depression among Indian women of childbearing age. Even water with low levels of As but within permissible limits in India showed a higher prevalence of depressive and neurobehavioral symptoms among women.
		The subjective symptoms questionnaire and Beck's 21-point depression inventory-II were used for NBS and depression detection, respectively.	Platelet activation and consequent secretion of alpha granules (e.g., P-selectin) and dense granule contents (e.g. serotonin) in the pathophysiology of depression among As-exposed women.	
Banerjee et al. (2012) *Cross-sectional study* (India)	952 female individuals	Depression	IAP; PM_2.5_; PM_10_; CO	Cooking with biomass was found to be an independent and strong risk factor for depression. The sample size of this study was large enough to conclude that the connection between chronic biomass smoke inhalation and depression in rural women was real rather than apparent. In essence, the study points to the potential danger of cooking with traditional biomass for the mental health of the rural womenfolk in India and other developing nations and advocates supply of cleaner fuel at an affordable cost to poor, rural families.
		BDI-II	Depleted platelet serotonin, suggesting altered serotonergic activity in the brain.	
Tomei et al. (2007) *Case-control study* (Italy)	607 Municipal Force Police employees and 306 traffic police officers	Depression, Anxiety	BaP (0.48 ng/m3), Polycyclic aromatic hydrocarbons (4.45 ng/m3), Lead (Pb) (22 ng/m3), Ni (9 ng/m3), CO (1.4 mg/m3), NO_2_ (77.5 g/m3), O_3_ (49.4 g/m3), benzene (4.35 g/m3), particulate matter 10 m in diameter or less (PM_10_) (50 g/m3) and sulfur dioxide (SO_2_) (4.7 ng/m3)	The chronic exposure to low doses of chemical stressor, interacting with and adding to the psychosocial ones, could affect DA plasma concentrations in traffic police officers of both sexes. In the long run, the increase in exposure levels and time could induce a reduction in DA levels.
		Questionnaire's items concerning anxiety, depression and panic attacks	Increased levels of DA due to urban pollutants exposure linked to depression or anxiety or panic attack	

4 HNE*: 4-hydroxy-2-nonenal;****ACTH****: adrenocorticotropic hormone;****APOE****: Apolipoprotein E;****BaP:****Benzo(a)pyrene;****BDI****: Beck Depression Inventory;****cAMP****: cyclic adenosine monophosphate;****CO****: carbon monoxide;****CO***_***2***_*: carbon dioxide;****CRP****: C-reactive protein****DA****: Dopamine;****dlPCBs****: dioxin-like Polychlorinated Biphenyls;****DNA****: Deoxyribonucleic acid;****F****: Female;****FST:****Forced Swim Test;****GDS-30:****Geriatric depression scale;****ESS:****Early Signs Scale;****HAM-D:****Hamilton Rating Scale for Depression****; HPA****: hypothalamic-pituitary-adrenal;****HPCBs****: higher Polychlorinated Biphenyls;****HVA****: Homovanillic acid;****IAP****: indoor air pollution;****IL-17****: Interleukin-17;****IL-6****: Interleukin-6;****L/DBT:****Light/Dark Box Test;****EPM:****Elevated Plus Maze;****LPCBs****: Low Polychlorinated Biphenyls;****M****: Male;****MBP****: mono-n-butyl phthalate;****MBzP****: mono-benzyl phthalate;****MeHg****: MethylMercury;****MEHP****: mono-2-ethylhexyl phthalate;****NBS****: Neurobehavioral symptoms;****NO***_***2***_*: nitrogen dioxide;****OFT:****Open Field Test;****PAE****: Phthalates;****PANSS****: Positive and Negative Symptom Scales;****PCB****: Polychlorinated and Hydroxylated Biphenyl;****PHQ-9:****Patient Health Questionnaire-9;****PM****: Particulate Matter;****SAS****: Self-Rating Anxiety Scale;****SDS****: Self-Rating Depression Scale;****SIT:****Social Interaction Test;****SMD****: severe mental disorders;****SO***_***2***_*: sulfur dioxide;****T4****: Thyroxine;****T-AOC****: total antioxidant capacity;****TTR****: Transthyretin;****VOCs****: Volatile organic compounds;****y****: year/years old;****YWHAB****: tyrosine 3-monooxygenase/tryptophan 5-monooxygenase activation protein beta*.

Particulates were the most investigated pollutants, In particular, 5 studies analyzed exposure to PM_2.5_ only (Yuan et al., 2023; Lei et al., 2023; Gao et al., 2021; Xu et al., 2024; Ahlers and Weiss, 2021); two studies investigated PM_10_ exposure together with other air pollutants, such as Benzo(a)pyrene, Polycyclic aromatic hydrocarbons, Nickel (Ni), Carbon Monoxide (CO), NO_2_, O_3_, Benzene, Sulfur Dioxide (Tomei et al., 2007) or CO, CO_2_, SO_2_, NO_2_, Volatile Organic Compounds (VOCs) (Abo-el-Ata et al., 2023); one study evaluated the exposure to both PM_2.5_ and PM_10_ (Wei et al., 2022); and two studies analyzed both PM_2.5_and PM_10_ exposure together with Arsenic (Mukherjee et al., 2014) or CO (Banerjee et al., 2012).

The remaining studies examined the exposure to Phthalates (one study out of 15), (Bao et al., 2022), to Polychlorinated and Hydroxylated Biphenyls (PCB) (two studies) (Gaum et al., 2017; Gaum et al., 2019), to pollutants resulting from industrial/military activity, gas extraction, nuclear material (one study) (Bjørklund and Semenova, 2023) and to Mercury (one study) (Ng et al., 2015).

13 studies out of 15 explored the potential mechanisms linking pollutants to depression, while the remaining three studies were focused on schizophrenia (Wei et al., 2022; Gao et al., 2021; Lei et al., 2023).

#### 3.1.1 Studies on exposure to PM_2.5_ and/or PM_10_

Yuan et al. (2023) examined PM_2.5_ deposited doses in three regions of the respiratory tract. The effects of acute exposure to PM_2.5_ and its deposits are related to an increase in health risks through higher blood pressure, increased anxiety and depressive symptoms, and modification of the urinary metabolomic profile by the activation of the cAMP signaling pathway (Yuan et al., 2023).

Some authors focused on the effects of pollutants’ exposure on women. Mukherjee et al. (2014) analyzed the exposure to PM_2.5_ and PM_10_ along with arsenic (As) in 342 premenopausal rural women living in West Bengal, India, compared with a control group of 312 women who were not drinking As-contaminated water. Authors found that women exposed to low levels of arsenic in drinking water for over 15 years showed higher rates of neurobehavioral symptoms and depression. Platelet activation and subsequent secretion of α-granule (e.g., P-selectin) and dense granule contents (e.g., serotonin) was noted as the pathophysiological mechanisms linking depression to pollutant exposure. Since the exposure levels to PM_2.5_ and PM_10_ were similar between cases and controls, the observed effects could be primarily attributed to arsenic exposure, being the sole pollutant with differing exposure levels between the two groups (Mukherjee et al., 2014). Similarly, Banerjee et al. (2012) evaluated exposure to both PM_2.5_ and PM_10_ along with CO (IAP - indoor air pollution - resulting from biomass burning) compared with a control group consisted of women not exposed to IAP; higher rates of depression and decreased platelet serotonin were found in women who cooked with biomass, a finding that may indicate altered serotonergic activity in the brain possibly linked to depressive symptoms. An increased expression of P-selectin on the platelet surface was also observed, indicating platelet hyperactivity with a consequent risk of cardiovascular disease (Banerjee et al., 2012). Ahlers and Weiss (2021) have shown that pregnancy may be associated with a specific vulnerability to PM_2.5_ exposure, which increases the risk of depression by activating the hypothalamic-pituitary-adrenal axis, as documented by increased cortisol levels. A greater prenatal exposure to PM_2.5_ is associated with more severe depressive symptoms experienced by the pregnant mothers in the third trimester of pregnancy. Furthermore, significant associations of higher PM_2.5_ exposure with both increased area under the cortisol curve (AUCG) and mean cortisol levels in women were observed. However, the cortisol parameters did not mediate the association between exposure to PM_2.5_ and depressive symptoms (Ahlers and Weiss, 2021).

Xu et al. (2024) found that metabolic risk factors (namely, hypertension, dyslipidemia, and diabetes), especially among the elderly, women, and people who do not drink alcohol, mediate the relationship between PM_2.5_ and depressive symptoms in 7794 participants (Xu et al., 2024).

Two studies (Wei et al., 2022; Gao et al., 2021) reported that PM exposure may increase the risk of relapse in schizophrenia, through different underlying mechanisms: decreased antioxidant system function, especially the level of T-AOC (Total Antioxidant Capacity) (Wei et al., 2022) and imbalance of inflammatory cytokine IL-17 (Gao et al., 2021). Lei et al. (2023) associated PM_2.5_ concentration with relapse in schizophrenia, which was also influenced by gender (with women at higher risk than men) and genetic factors, in particular the polymorphism of the YWHAB gene (locus rs6031849). Particularly, high long-term levels of PM_2.5_ exposure increase the risk of recurrence of schizophrenia (Lei et al., 2023).

Other authors found a relationship between work-related PM exposure and non-psychotic psychiatric conditions, like depression. In particular, Tomei et al. (2007) explored the possible relationship between occupational exposure to urban chemical pollutants (including PM_10_, Benzo(a)pyrene, Polycyclic Aromatic Hydrocarbons, Ni, CO, NO_2_, O_3_, Pb, Benzene and Sulfur Dioxide), dopamine (DA) plasma levels and related psychopathological conditions in 306 traffic policemen compared to 301 control subjects. The chronic exposure to these low-dose professional chemical stressors, in combination with psychosocial stressors, could change DA plasma levels, which were significantly higher in traffic police officers compared to the control group. These data suggest a possible link between urban chemical stressor exposure, plasma DA levels, and the onset of anxiety and depressive symptoms (Tomei et al., 2007).

Abo-el-Ata et al. (2023) evaluated the effects of occupational exposure to air pollutants (including PM_10_, CO, CO_2_, SO_2_, NO_2_, VOCs) on the mental health of pottery workers. In addition, they assayed serum levels of 4-hydroxy-2-nonenal (4 HNE), an oxidative stress marker. They found that workers exposed to air pollutants, particularly pottery workers, had impaired neuropsychological functions and a higher risk of depression. The increase in serum 4 HNE levels associated with exposure to air pollutants may contribute to both altered neuropsychological function and increased risk of depression (Abo-el-Ata et al., 2023).

#### 3.1.2 Studies on exposure to PCB (Polychlorinated and Hydroxylated Biphenyls)

Polychlorinated biphenyls (PCBs) are a group of man-made chemicals, which were commonly used in many kinds of building materials and electrical products before 1980. PCBs are contained in caulk, paints, glues, plastics, fluorescent lighting ballasts, transformers, and capacitors (Polychlorinated Biphenyls).

Two separate studies by Gaum et al. (2017, 2019) found that exposure to PCBs may lead to depressive symptoms through an altered dopamine metabolism. In particular, in the 2019 study, the authors explored the relationship of PCBs with free thyroxine (fT4) in a sample of 116 participants and found that occupational and inhalative PCB exposure might exert a depressive effect via a physiological process in the DA and thyroid systems (Gaum et al., 2019). In the 2017 study, increased PCB exposure was associated with stronger depressive symptoms and changes in the dopamine metabolism across time in 178 individuals (Gaum et al., 2017).

#### 3.1.3 Studies on other pollutants

##### 3.1.3.1 Phthalates

Bao et al. (2022) explored the relationship between phthalates exposure and depressive symptoms in 989 participants aged 60 and above. The authors found that higher urinary concentrations of certain phthalates (MEHP - Mono (2-ethylhexyl) phthalate, MBzP - Mono benzyl phthalate, and MBP - Mono butyl phthalate) were more likely to be associated with depressive symptoms in elderly people. Furthermore, they found that the relationship between MEHP exposure and increased risk of depressive symptoms is only partially mediated by chronic inflammation (Bao et al., 2022).

##### 3.1.3.2 Pollution resulting from industrial or military activity

The potential effects of environmental pollution on stress hormone levels and depression in those exposed to industrial and military pollution (deriving from non-ferrous industry, condensate gas extraction, and radioactive fallout from nuclear weapons tests) have been recently proposed (Bjørklund and Semenova, 2023). Individuals living in polluted areas have higher median serum cortisol concentrations and lower median serum ACTH levels, as well as a higher prevalence of primary hypoadrenalism. Most importantly, the inhabitants of the two control sites have a lower median score at the Patient Health Questionnaire-9 **(**PHQ-9**)** compared to those living in polluted areas, indicating fewer depressive symptoms and highlighting the significant impact of environmental pollution on the levels of stress hormones and on the risk of depression (Bjørklund and Semenova, 2023).

Ng et al. (2015), evaluating the role of genetic factors and environmental toxins in child development on 166 mother-infant pairs, suggested that the increased levels of mercury in cord blood may be one of the risk factors for deficit behavior in preschool aged children, particularly among those carrying the APOE e4 allele. In fact, they found that children carrying the APOE e4 allele who had higher mercury levels in their cord blood had increased indicators of behavioral deficits, as measured by the Child Behavior Checklist (CBCL), including symptoms of anxiety and depression. This suggests that high mercury exposure in utero might increase the risk of developmental problems in genetically predisposed children with the APOE e4 allele (Ng et al., 2015).

**Fig. 2:**
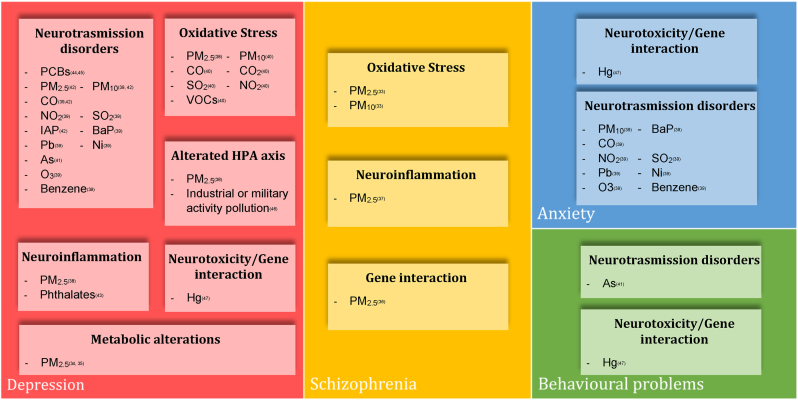
Clinical meaning of pollutants molecular targets-HUMAN STUDIES.

Fig. 2 summarizes the links between pollutants, mental disorders, and biological mechanisms in humans.

### 3.2 Studies on animal models

The majority of the 31 studies were conducted on rodents: 19 on mice, and 7 on rats; four studies were conducted on zebrafishes, and one study was conducted on Mauremys sinensis turtles (Table 2). 14 papers did not explicitly mention the exact number of animals included in the analyses. The sample size of the remaining studies ranged from 15 (Xie et al., 2024) to 192 animals (Mokoena et al., 2010), with a total sample of 1055. Not all papers further specified **sex** distribution.

**Table 2:** Studies involving animal models.

Author (year) *type of study (Country)*	Population	SMD	Pollutant	Main findings
		Assessment	Mechanism	
Choi et al. (2024) *Experimental study (Republic of Korea)*	Preadolescent C57BL/6 mice exposed to PM_10_	Schizophrenia-like behaviors	PM_10_	PM_10_ exposure in preadolescence exacerbates cognitive impairment in the MK-801-induced schizophrenia model, likely facilitated by decreased BDNF levels through reduced ERK-CREB expression.
		Behavioral tests (OFT, Social interaction test, NOR test, Y-maze test)	PM_10_ exposure reduced the expression levels of brain-derived neurotrophic factor (BDNF) and phosphorylation of ERK-CREB signaling pathway in the hippocampus, exacerbating schizophrenia-like behaviors	
Debler et al. (2024) *Experimental study (USA)*	Female C57BL/6 N mice, 8 per group	Depressive-like behavior	TCDD (2,3,7,8-tetrachlorodibenzo-p-dioxin)	TCDD induced a depression-like phenotype characterized by reduced sucrose preference, increased immobility in FST, and decreased grooming time. Effects were not due to gross lethal toxicity.
		SPT, Splash Test, FST, Morris Water Maze	Activation of Aryl Hydrocarbon Receptor (AhR)	
Ma et al. (2024) *Experimental study (China)*	32 Male BALB/c mice,	Depressive-like behavior, Anxiety-like behavior, social deficits	Nanoplastics (NPs) Surface-modified polystyrene nanoparticles (PS, PS-COOH, PS-NH_2_)	PS-NPs accumulated in the brain. These nanoplastics entered the brain by either disrupting the blood-brain barrier or bypassing it through endocytosis and macropinocytosis pathways. All PS-NPs targeted mitochondria, resulting in increased mitochondrial reactive oxygen species (mROS), decreased mitochondrial membrane potential, and diminished ATP levels. This exposure caused neuronal damage, apoptosis, and neuroinflammation, as indicated by the reaction of astrocytes and microglia in the mouse brain.
		OFT, EPM, social preference, novelty, Y-maze and FST	Mitochondrial dysfunction in neurons oxidative stress, neuroinflammation	
Wang et al. (2024) *Experimental study (China)*	Zebrafish, 40 per treatment group	Depressive-like behavior	Sodium p-perfluorinated nonenoxybenzene sulfonate (OBS)	OBS reduces mitochondrial membrane potential, causing fragmentation. This fragmentation results in mitochondrial dysfunction and an overproduction of reactive oxygen species (ROS). The excessive ROS, along with elevated levels of inflammatory factors such as IL-1β, TNF-α, NF-κB, and IL-6, significantly amplifies the inflammatory response. This cascade ultimately leads to cell apoptosis, neurotoxicity, and behavioral abnormalities.
		CPP, Social Interaction (SI), T-maze Test	Mitochondrial dysfunction, oxidative stress, neuroinflammation	
Wu et al. (2024) *Experimental study (China)*	70 Mauremys Sinensis turtles	Depressive-like behavior	Butyl paraben (BuP)	The depressive-like behaviors in the turtles under BuP stress may be regulated via an increase in the 5-HT rather than DA, also via an increase of GABA-T and tyrosine hydroxylase activity and a decrease of AChE activity in the brain. The increase of 5-HT is associated with higher mRNA expression levels of 5-HTR, 5-HT transporter (Drd4, Slc6a4) and TPH. BuP stress also causes cell apoptosis in the brain of the higher BuP exposed groups.
		Behavior observation: basking, feeding; body righting, movement speed, altering.	Neurotransmission and brain cell apoptosis	
Xie et al. (2024) *Experimental study (China)*	15 Kunming SPF mice (5 male and 10 female)	Depressive-like behavior	Diisodecyl phthalate (DIDP) and O_3_	Prenatal co-exposure to DIDP and O_3_ exacerbated depressive-like behaviors in offspring mice, with oxidative stress and TWIST1 involvement. Vitamin E supplementation alleviated these effects.
		Behavioral tests (FST, Tail suspension test, OFT, SPT)	DIDP and O_3_ exposure led to oxidative stress, HPA axis activation, and changes in TWIST1 expression, contributing to depressive-like behaviors	
An et al. (2023) *Experimental study (China)*	64 C57BL/6J mice	Depressive-like behavior Anxiety-like behavior	Perfluorooctane sulfonate (PFOS)	PFOS exposure may inhibit the synaptic plasticity of the hippocampus via glutamatergic synapse and the CREB/BDNF signaling pathway to cause depressive-like behaviors in male mice.
		OFT; EPM test; FST; TST; NSF test	Neuronal plasticity via activation of the glutamatergic synapse and inhibition of the downstream CREB/BDNF signaling pathway	
Baumann et al. (2023) *Experimental study (USA)*	- BALB/c mice - CD-1 mice - SAA 1/2/3 triple knockout mice	Depressive-like behavior	O_3_	Circulating SAA, lung inflammation, Ido1 mRNA expression and kynurenine were increased after 3 ppm O_3_, whereas depressive-like behaviors are observed at all O_3_ doses. This suggests that behavioral responses to O_3_ can occur independently of increased SAA or neutrophils in the lungs. Using SAA knockout mice, it was found SAA did not contribute to O_3_-induced pulmonary damage or inflammation, systemic increases in kynurenine post-O_3_, or depressive-like behavior.
		SPT; OFT; TST	Lung-brain axis mechanism: circulating factors (SSA A and kynurenine) upregulated in response to pulmonary inflammation have neuromodulatory functions, associated with depression	
Cao et al. (2023) *Experimental study (China)*	Zebrafish larvae	Depressive-like behavior	2,2′,4,4′-tetrabromodiphenyl ether (BDE-47)	BDE-47 exposure disrupted the NIF visual pathway (which mainly depends on the ipRGCs that employ melanopsin as the signaling photopigment) and resulted in depression-like effects.
		Thigmotaxis Test; Sleep/Wake Behavior	NIF visual system-mediated neurotoxic mechanism	
Huo et al. (2023) *Experimental study (China)*	Zebrafishes	Depressive-like behavior	Sulfonamides	Exposure to Sulfonamides at environmentally relevant concentrations induces developmental and neurotoxic effects in zebrafish, impacting folate synthesis pathways and carbonic anhydrase metabolism.
		Behavioral measurement of zebrafish: total distance traveled, speed, acceleration, moved state, activity average value, activity and 10-min dark behavior experiment	Downregulation or inhibition of key genes involved in folate synthesis and carbonic anhydrase metabolism	
Liu et al. (2023) *Experimental study (China)*	Zebrafish adults	Depressive-like behavior Anxiety-like behavior Cognitive function Sociability	Imidacloprid	Constant exposure of imidacloprid significantly induced behavioral disorders in zebrafish adults, associated with strong transcriptional changes leading to disruption of circadian rhythm, metabolic imbalance of arginine and proline, and neurotransmitter disorders.
		Novel tank test (anxiety-like behavior); T-maze test (learning and memory behavior); social preference test (social behavior)	Transcriptional changes	
Shin et al. (2023) *Experimental study (Korea)*	70 offspring mice 20 embryos	Depressive-like behavioral Anxiety-like behavior Cognitive function Nesting behavior Sociability	Polystyrene particles	Exposure to Polystyrene particles decreases Gabra2 expression and increases depression-like behaviors.
		FST (depression); TST (depression); OFT; EPM test (anxiety); Three-chamber social test (sociability); NOR (sociability); Morris water maze (cognitive); Nest building test (nesting)	Gabra2 gene expression	
Ehsanifar et al. (2022) *Experimental study (Iran)*	24 NMRI mice	Anxiety-like behavior Depressive-like behavior Cognitive functions (memory and learning)	Diesel exhaust particles (DEPs)	Chronic exposure to DEPs leads to inflammatory markers upregulation and changes in hippocampal structure (reduction of the density of neurons in hippocampus CA1, CA3, and dentate gyrus regions) resulting in neurobehavioral alteration and affective and cognitive impairment in male mice.
		- OFT (anxiety) - EPM test (anxiety) - FST (depressive) - Barnes maze test (memory and learning)	Neuroinflammation and change in neuronal morphology.	
Ji et al. (2022) *Experimental study (China)*	80 C57BL/6 mice	Depressive-like behavior	PM_2.5_	PM_2.5_, via both particle translocation and systemic inflammation, caused microglia activation wich is characterized by excessive release of TNFα, and induced TNFα-mediated caspase3 apoptosis pathway, leading to neuronal death in olfactory bulb with depression-like behaviors in mice.
		OFT	Microglia activation-apoptosis of cells of olfactory bulb	
Kochi et al. (2021) *Experimental study (USA)*	577 rats	Anxiety-like behavior Depressive-like behavior Cognitive functions (memory)	Pro-oxidants CO_2_, NO_2_ and CO (simulated SVEE)	SVEE causes activation of the oxido-inflammatory processes which collectively reduce BDNF protein expression, causing suppression of NMDA receptor subunit expression. Suppression of the PKC, ERK 1/2, CaMKIV-BDNF pathway is consequential in terms of disrupting neurogenesis and markers of synaptic plasticity, together causing behavioral and cognitive deficits in male rats.
		- OFT (anxiety) - L/DBT (anxiety) - EPM test (anxiety) - Y-maze test (memory function) - FST (depression)	Oxido-inflammatory process	
Li et al. (2021) *Experimental study (China)*	100 mice	Anxiety-like behavior Depressive-like behavior	NO_2_	NO_2_ exposure could cause anxiety and depression-like disorders in adult mice in a sex-dependent manner, with male mice being more susceptible to NO_2_ than female mice. The structural and functional abnormalities of myelin sheaths in male mice could contribute to the specificity of neurobehavioral disorders caused by NO_2_ exposure. Additionally, prolactin might play a critical role in the sex-specific damage to myelin sheath and mental disorders.
		- OFT (anxiety) - EPM test (anxiety) - MB test (anxiety) - FST (depression)	Morphology-inflammatory process	
Wang et al. (2021) *Experimental study (China)*	32 mice	Depressive-like behavior Anxiety Emotional disorders	MCA	MCA exposure: - thyroid dysfunction - influence on DAT expression in the striatum - changed locomotive activity, anxiety-like and depressive-like behaviors
		OFT; L/DBT; FST; Circadian activity test	- HPT disfunction - striatum dopamine alteration	
Xu et al. (2021) *Experimental study (China)*	Wild type and ApoE mice	Depressive-like behavior	Copper (Cu)	Copper exposure exacerbates depression-like behavior of ApoE4 mice and the mechanisms may involve the dysregulation of synaptic function and immune response and overactivation of neuroinflammation
		EPM testFST; Morris water maze	Dysregulation of synaptic function and immune response and overactivation of neuroinflammation.	
Dridi et al. (2021) *Prospective cohort study (France)*	Offspring mice from 40 pregnant mice	Depressive-like behavior	POPs (including organochlorine pesticides, PCBs, PAHs, BDEs, polychlorinated dibenzo-p-dioxins/dibenzofurans (PCDD/Fs)), metals including mercury (Hg) and lead (Pb)	Offspring males perinatally exposed to naturally contaminated reared and river eels with persistent organic pollutants (POPs) and heavy metals displayed chronic depression-like phenotype.
		TST FST	oxidative stress	
Salvi et al. (2020) *Cohort study (USA)*	SD rats	Anxiety- and depressive-like behavior	Simulated SVEE:pro-oxidant constituents of vehicle exhaust such as CO_2_, CO and NO_2_	SVEE is a toxicological stressor that induces oxidative stress and results in mitochondrial impairment, which by disrupting neural circuitry impairs cognitive and behavioral functions.
		OFT; EPM test; L/DBT; MB test; Depression-like behavior test	Neurotoxicity	
Chu et al. (2019) *Prospective cohort study (China)*	60 mice	Depressive-like behavior	PM_2.5_	Depressive-like responses were caused by ambient PM_2.5_dose-dependently in rats and mice, which might partly attribute to the disorders of neurotransmitters. After PM_2.5_exposure for 9 weeks in mice and 12 weeks in rats, the significant inflammatory changes and oxidative stress had been observed.
		OFT; NSF test; SPT; Mouse TST	toxic elements deposition, oxidative stress and the inflammation in prefrontal cortex and regulating role of Nrf2/NLRP3 signaling pathway modulating inflammation	
Ehsanifar et al. (2019) *Longitudinal cohort study (Iran)*	48 adult NMRI mice	anxiety and depression-like behavior	Nanoscale diesel engines exhausted particles (DEPs)	The different times of DEPs exposure, leads to oxidative stress and inflammatory in plasma and brain regions. That this cumulative transport of inhaled nanoscale DEPs into the brain and creating to inflammation responses of brain regions may cause problems of brain function and anxiety and depression.
		EPM and FST	induction of MDA and nitrite oxide (NO) in brain regions and neuronal nitric oxide synthase (nNOS) mRNA followed by IL6, IL1α, and TNFα.	
Raeis-Abdollahi et al. (2019) *Experimental study (Iran)*	Offspring adults of 28 pregnant dams.	Anxiety-like and Depressive-like behavior	Chrysotile asbestos	prenatal exposure to Chrysotile asbestos → oxidative stress during pregnancy: - hippocampus alterations in male offspring: - reduction of cellular proliferation - reduction of neuronal maturation - dentate gyrus astrogliosis - depression-like behaviors, high anxiety levels in male offspring
		EPM FST	- oxidative stress - lipid peroxidation (MDA) - pro-oxidants/antioxidants unbalance - hippocampal alterations	
Jia et al. (2018) *Experimental study (China)*	Human model: 12 non-smoking male adults. Mice model: 20 C57 BL/6 J male mice	Depressive behaviors	PM_2.5_	A causal relationship between PM exposure and mental disorder was reported. Heavy particulate pollution has the possibility to aggravate the symptoms of mental illness, and the risks of mood-related behaviors disorder in human.
		Humans: no test Mices: OFT, L/DBT, EPMT, FST, and SIT	Neurotoxicity, and impaired the neuron structure in mice hippocampus	
Liu et al. (2018) *Experimental study (China)*	C57BL/6 mice	Depressive like behaviors	PM_2.5_	Continuous high-level PM exposure alters the depressive-like response in mice and induces a damage-repair-imbalance reaction.
		Behavioral tests: FST; - Mouse TST;	Systemic inflammation	
Lestaevel et al. (2015) *Case-control study (France)*	38 offspring rats from 42 pregnant Sprague-Dawley rats	Depression, Anxiety	Uranium	Exposure to Uranium from birth to 10 weeks induces behavioral and neurochemical consequences.
		OFT; EPM; Rotarod test; Porsolt test (FST).	Oxidative stress	
Xu et al. (2015) *Experimental study (China)*	Offspring of 60 pregnants ICR mice	Depression-like and Anxiety-like behaviors	DEHP	Perinatal exposure to DEHP affected anxiety- and depression-like behaviors in offspring mice, with significant changes in open arm entries and time spent in open arms. DEHP exposure altered the expression of AR and ERb in the hippocampus, suggesting a potential role of gonadal hormones in modulating these behaviors.
		OFT; dark/light transition; mirrored maze; EPM; FST	- Estradiol/Testosterone - Hippocampus receptor expression: AR, ERb gonadal hormones → MAPK/ERKs signaling pathway in hippocampus	
Ye et al. (2015) *Case-control study (China)*	30 Sprague-Dawley rats	Depressive-like behavior	Tris-(2,3-dibromopropyl) isocyanurate (TDBP-TAZTO)	Six-month exposure to TDBP-TAZTO induced neurotoxicity on adult rat hippocampal neurons, increased inflammatory and oxidative stress markers, overexpression of pro-apoptotic proteins, reduced expression of neurogenesis-related proteins, and damage to hippocampal neurons in DG, CA1, and CA3 areas. These effects may contribute to cognitive impairment and depression-like behaviors.
		OFT (Locomotor Activity); FST (Depression-like behavior); Morris Water Maze Test	inflammatory and oxidative stress	
Zuo et al. (2014) *Experimental study (China)*	64 BALB/c mice	Depression-like behavior	Di-(n-butyl)-phthalate (DBP)	The development of an atopic allergy in mice may increase depressive-like behaviors; this effect could be potentiated by the presence of DBP in the model.
		FST; TST; OFT	Di-(n-butyl)-phthalate-induced Oxidative Stress and its link to Depression-like Behavior in Mice with or without Ovalbumin Immunization	
Davis et al. (2013) *Experimental study (USA)*	Adult C57BL/6J	Depressive like behavior	nPM	Prenatal exposure to nPM is consistent with neurodevelopmental abnormalities in humans exposed to airborne pollution from vehicular traffic
		OFT and EPM	Neurotoxicity	
Fonken et al. (2011) *Experimental study (USA)*	C57BL/6 mice	Depressive like behavior, learning and memory impairment	PM_2.5_	Ten months of exposure to airborne fine particulate matter leads to upregulation of inflammatory markers and hippocampal structural changes resulting in depressed-like affective responses and cognitive impairment in mice.
		FST, Barnes maze	Oxidative stress	
Mokoena et al. (2010) *Experimental study (South Africa)*	192 Sprague Dawley rats	Depression	O3	O_3_ does not affect behavior in stress-naïve animals but attenuates the antidepressant-like effect of the antidepressant Imipramine. Ozone furthermore induces an increase in hippocampal oxidative stress and oxidative damage, of which only the oxidative damage induced by chronic ozone exposure is reversed by Imipramine.
		FST	Hippocampal superoxide and MDA levels	

***5-HT****: 5-Hydroxytryptamine;****5-HTR****: 5-Hydroxytryptamine receptor;****AChE****: acetylcholinesterase;****APOE****: Apolipoprotein E;****AR****: Androgen Receptor;****BDE****: Bromodiphenyl Ether;****BDE-47****: 2,2′,4,4′-tetrabromodiphenyl ether;****BDNF****: brain-derived neurotrophic factor;****BuP****: Butyl paraben;****CA****: cornus ammonis;****CA1****: Cornus ammonis 1;****CA3****: Cornus ammonis 3;****CaMKIV****: Calcium/calmodulin-dependent kinase IV;****CAT****: Catalase;****CO***_***2***_*: carbon dioxide;****CPP:****Conditional Position Preference;****CREB****: cyclic adenosine monophosphate-responsive element-binding protein;****DA****: Dopamine;****DAT****: Dopamine Transporter;****DEHP****: di-(2-ethylhexyl) phthalate;****DEPs****: diesel engines exhausted particles;****DG****: dentate gyrus;****Drd4****: Dopamine Receptor D4;****EPM:****Elevated plus maze;****ERb****: Estrogen receptor beta;****ERK****: extracellular signal-regulated kinases;****ERKs****: Extracellular signal-regulated kinases;****F****: Female;****FST:****Forced Swim Test;****GABA-T****: GABA transaminase;****Gabra2****: Gamma-aminobutyric acid receptor subunit alpha-2;****HPT****: Hypothalamic–pituitary–thyroid axis;****Ido1****: indoleamine 2,3-dioxygenase;****ipRGCs****: intrinsically photosensitive retinal ganglion cells;****L/DBT****: Light/Dark Box Test;****m****: month/months old;****M****: Male;****MB:****Marble burying;****MAPK****: Mitogen-activated protein kinase;****MCA****: Melamine Cyanuric Acid;****MDA****: Malondialdehyde;****mRNA****: messenger Ribonucleic acid;****NLRP3****:nucleotide-binding domain, leucine-rich–containing family, pyrin domain–containing-3;****Nrf2****:Nuclear Factor E2-related Factor-2;****NIF****: non-image-forming;****NMDA****: N-Methyl-D-aspartate receptor;****NO***_***2***_*: nitrogen dioxide;****nPM****: nanoscale particulate matter;****NOR:****Novel object recognition;****NSF:****Novelty suppressed feeding;****O***_***3***_*: ozone;****OFT:****Open field test;****OVA:****Ovalbumin****PAHs****: Polycyclic Aromatic Hydrocarbons;****PCBs****: Polychlorinated Biphenyls;****PKC****: Protein kinase C;****PM****: Particulate Matter;****PND****: Post Natal Days;****POPs****: persistent organic pollutants;****ppm****: parts* per *million;****Slc6a4****: Solute carrier family 6 member 4;****SIT:****Social interaction test****SOD****: Superoxide Dismutase;****SPT:****Sucrose preference test;****SSA****: Serum Amyloid A;****SVEE****: vehicle exhaust exposure;****TST:****Tail suspension tes****t; TNFα****: Tumor necrosis factor α;****TPH****: tryptophan hydroxylase;****w****: week/weeks old.*

Depression-like behaviours were considered in 21 studies while seven considered depression-like and anxiety-like behaviours; two studies considered depression-like and anxiety-like behaviors and memory together with social preferences or learning abilities. Only one study out of 31 focused on schizophrenia-like behaviors (Choi et al., 2024).

The most frequently investigated pollutants in studies on animal models were PM_2.5_ (4 studies), O_3_ (3studies), and pro-oxidants agents: NO_2_, CO_2_, CO (2 studies). Other pollutants were: Persistent Organic Pollutants; Tris-(2,3-dibromopropyl) Isocyanurate; Uranium; Di-(n-butyl)-Phthalate; Butyl Paraben; Sulfonamides; Perfluorooctane Sulfonate; Polystyrene particles; 2,2′,4,4′-Tetrabromodiphenyl Ether; Diisodecyl phthalate; 2,3,7,8-tetrachlorodibenzo-p-dioxin; Imidacloprid; Diesel Exhaust Particles; Copper; Melamine Cyanuric Acid; Chrysotile Asbestos; Di-(2-ethylhexyl)-Phthalate; PM_10_; Sodium p-perfluorinated noneoxybenzen sulfonate; Nanoscale Diesel engines Exhausted Particles; Nanoplastics; nano PM.

#### 3.2.1 Studies on nano PM**,** PM2.5 and/or PM10 exposure in animal models

Chu et al. (2019), Liu et al. (2018), Ji et al. (2022), and Fonken et al. (2011) have shown different adverse outcomes, including significant inflammatory changes, oxidative stress, depressive-like behaviors, cognitive impairment, and structural hippocampal changes following exposure to PM_2.5_ in rodents. In particular, Chu et al. (2019) identified the Nrf2/NLRP3 signaling pathway (which modulates inflammation) as key regulator of depression associated to PM_2.5_ exposure, reporting that depressive-like responses to PM_2.5_ were dose-dependent and could be partly explained by dysfunctions in neurotransmitter systems and the accumulation of toxic elements from polluted air, such as Be, Al, Cr, Co, Ni, Se, Cd, Ba, Ti, and Pb. Fonken et al. (2011) reported that 10 months of exposure to airborne fine particulate matter ( < 2.5 μm (PM_2.5_)) resulted in an upregulation of inflammatory markers and structural hippocampal changes, leading to depressed-like emotional responses and cognitive impairment in mice (Chu et al., 2019; Fonken et al., 2011). Continuous exposure to high PM levels can lead to depressive-like behaviors in mice and to abnormal response in depressive-like behaviors by the disturbance of a balance between neuronal damage and repair processes (Liu et al., 2018). Finally, Ji et al. (2022) explored the effects of inhaled PM_2.5_ on the olfactory bulb (OB) in mice, with a focus on behavioral alterations, microglial morphology, neuronal death, and inflammation. Microglial activation, identified as a hallmark of PM_2.5_ neurotoxicity in the OB, may provide vital information regarding the early markers and mechanisms involved in depression associated with PM_2.5_ exposure (Ji et al., 2022).

**T**he only study investigating PM_10_ exposure in an animal model was also the only one to examine schizophrenia-like behaviors. Specifically, the study by Choi et al. (2024), investigated the effects of PM_10_ in combination with MK-801, an N-methyl-D-aspartate (NMDA) receptor antagonist known to induce schizophrenia-like symptoms. Mice exposed to PM_10_ showed exacerbated schizophrenia-like symptoms, including hyperlocomotion, anxiety, social interaction deficits, and cognitive impairments during adolescence and adulthood. PM_10_ exposure decreased the expression in the region of the hippocampus of Brain-Derived Neurotrophic Factor (BDNF), which plays a crucial role in memory consolidation and cognitive function; its reduction is linked to schizophrenia's cognitive impairments. PM_10_ exposure impaired the activation of the ERK-CREB signaling pathway, which is also involved in regulating BDNF expression. This disruption in signaling contributes to cognitive dysfunction and exacerbates the effects of MK-801-induced schizophrenia-like behaviors (Choi et al., 2024). Lastly, Davis et al. (2013) noted that prenatal exposure to nanoscale particulate matter (nPM) impairs neuron differentiation and causes sex-specific depressive-like behavioral changes in adult female mice (Davis et al., 2013).

#### 3.2.2 Studies on **O**_**3**_ exposure in animal models

Baumann et al. (2023) found that exposure to 3 parts per million (ppm) of O_3_ increases circulating serum amyloid A (SAA), indoleamine 2,3-dioxygenase (Ido1) mRNA expression, and kynurenine levels, and causes lung inflammation, while depressive-like behaviors were observed at all tested O_3_ doses (1–3 ppm). These behavioral changes were independent from SAA and neutrophil lung levels. Subsequent studies with SAA knockout mice, however, demonstrated that O_3_-induced lung injury, inflammation, systemic increases in kynurenine, or depressive-like behaviors were not mediated by SAA (Baumann et al., 2023). On the other hand, Mokoena et al. (2010) found that the exposure to O_3_ did not influence behavior in rats not previously exposed to stress but reduced the antidepressant-like effects of imipramine. Additionally, in the same animal model, O_3_ exposure increased hippocampal oxidative stress and damage, but treatment with imipramine reduced only the damage from chronic O_3_ exposure (Mokoena et al., 2010).

#### 3.2.3 Studies on pro-oxidants agents - simulated vehicle exhaust exposure (SVEE) in animal models

Kochi et al. (2021) and Salvi et al. (2020) assessed the effect of pro-oxidant agents, such as NO_2_, CO_2_ and CO on rats after exposure to simulated vehicle exhaust (SVEE), and found that exposure to pro-oxidant agents led to memory deficits in male rats but not in females, and that the male rats also exhibited anxiety- and depression-like behaviors (such as apathy, despair, irritability, social aversion, cognitive impairment and alterations of psychomotor activity, feeding and sleep) (von Mücke-Heim et al., 2023). Concomitantly, high levels of serum corticosterone, oxidative stress, and inflammatory markers (CRP and TNFα), and low levels of total antioxidant capacity and activities of glutathione, glyoxalase, and superoxide dismutase (SOD) were found. Additionally, a brain region-specific downregulation of Cu/Zn SOD, Mn SOD, GSR, PKCα, ERK1/2, CaMKIV, CREB, BDNF and NMDAR subunit protein expression (all related to oxidative stress response and neuronal signaling) was observed in male rats (Kochi et al., 2021).

Salvi et al. (2020) also reported that SVEE caused anxiety- and depression-like behaviors in rats following increased oxidative stress, reduced antioxidant response, and mitochondrial impairment. This was further confirmed by disturbance in the electron transport chain, lower oxygen consumption, reduced ATP synthesis, and changes in the dynamics of mitochondrial biochemicals (Salvi et al., 2020).

#### 3.2.4 Studies on diesel exhaust particles (DEPs) in animal models

Ehsanifar et al. (2019; 2022) published two studies on the *in vivo* impact of diesel exhaust particles (DEPs) in mice. The former study aimed at evaluating the impact of a nanosized subfraction of diesel engine exhaust particulate matter (DEPs <200 nm) in adult male mice, reporting that DEPs exposures caused oxidative stress and inflammation both in plasma and brain tissues, pointing out that cumulative inhaled nanoscale DEP transport to the brain may result in cerebral dysfunction, anxiety, and depression through inflammatory responses in the brain (Ehsanifar et al., 2019). In the 2022 study, authors found that DEPs-exposed mice had more severe disturbances concerning spatial memory and learning, along with depressive-like behaviors compared to not-exposed mice. In addition, there was an increase in the expression of hippocampal pro-inflammatory cytokines in DEPs-exposed mice, and a noticeable decrease in the density of neurons within the hippocampus CA1, CA3, and dentate gyrus (DG) regions (Ehsanifar et al., 2022).

#### 3.2.5 Studies on phthalates in animal models

Phthalates are primarily utilized as softeners to enhance the flexibility and durability of plastics. Three studies focused on the impact of phthalate exposure on mental health and the underlying mechanisms in mice.

Xu et al. (2015) found that perinatal exposure to Di-(2-ethylhexyl)-Phthalate (DEHP) enhances anxiety and depression-like behaviors in offspring mice, potentially interfering with gonadal hormone activity through alterations in the hippocampal expression of both androgen receptor (AR) and estrogen receptor beta (ERb) (Xu et al., 2015). Notably, in a previous study, Zuo et al. (2014) highlighted a complex mechanism linking phthalates, depression-like behaviours and atopic allergy in mice, examining the role of Di-(n-butyl)-Phthalate (DBP) exposure and its interaction with Ovalbumin (OVA). Atopic allergies can increase the risk of depression in mice, and DBP can facilitate the effects of OVA. Authors also reported an association between brain oxidative stress and pathogenesis of depression (Zuo et al., 2014).

In addition, a recent study by Xie et al. (2024), investigated the combined effects of prenatal exposure to diisodecyl phthalate (DIDP) and O₃ on the development of depressive-like behavior in offspring mice. Findings showed that prenatal exposure to both DIDP and O₃ increased depressive-like behaviors in offspring mice compared to either DIDP or O₃ alone. The co-exposure significantly elevated stress-related hormones (CRH, ACTH, cortisol) and reduced critical neurotransmitters like serotonin (5-HT), dopamine (DA), and norepinephrine (NE), which are linked to mood regulation. The combined exposure caused increased oxidative stress in the brain, which plays a crucial role in developing depressive symptoms, as also shown by the transcription factor TWIST1, associated with stress responses, implicated in these behavioral and molecular changes (Xie et al., 2024).

#### 3.2.6 Studies on heavy metals and related compounds in animal models

A chronic depression-like condition following long-term exposure to persistent organic pollutants (POPs) and heavy metals was found in male mouse offspring, who were perinatally fed with contaminated eels with high levels of POPs and heavy metals. Increased levels of superoxide dismutase (SOD) and catalase (CAT) were found in the hippocampus, indicating a response to oxidative stress. However, no significant changes in lipid peroxidation, measured by malondialdehyde (MDA) levels, were found in the hippocampus, prefrontal cortex, and cerebellum, suggesting that oxidative stress-related disturbances might be minimal in middle-aged brains despite the chronic depression-like behavior (Dridi et al., 2021).

Other studies have explored specific heavy metals. Lestaevel et al. (2015) found that uranium exposure in early development stages induces anxiety and depressive-like behaviors in offspring rats mediated by lipid peroxidation and oxidative stress (Lestaevel et al., 2015); Xu et al. (2021) showed that low-dose Cu exposure exacerbates depression-like behaviors in ApoE4 gene-carrying mice, with the involvement of neuroglial cells and various neurological pathways, including the Ras signaling pathway, protein export, axon guidance, and serotonergic, GABAergic, and dopaminergic synapses (Xu et al., 2021).

#### 3.2.7 Studies on other pollutants linked to depression in rodent models

Debler et al. (2024) investigated depressive-like symptoms in female mice exposed to 2,3,7,8-tetrachlorodibenzo-p-dioxin (TCDD), a prototypical high-affinity ligand of the aryl hydrocarbon receptor (AhR) and a standard reference for evaluating AhR activity. The study revealed that TCDD exposure led to reduced sucrose preference in the Sucrose Preference Test (SPT), indicative of anhedonia in rodents, and increased immobility in the forced swim test. However, spatial learning remained unaffected, suggesting TCDD's specific influence on mood regulation rather than cognitive impairment, potentially through AhR activation (Debler et al., 2024). Ma et al. (2024) evaluated the accumulation on nanoplastics (NPs), specifically polystyrene nanoplastics (PS-NPs) with different surface modifications (PS, PS-COOH, and PS-NH_2_), in mouse brains. The NPs were found to enter the brain by disrupting the blood-brain barrier or surpassing it via the endocytosis and macro-pinocytosis pathways. NPs exposure induced serious alterations in the behavioral tests, including anxiety- and depression-like changes, and decreased social interactions in the exposed subjects compared to the control mice. All PS-NPs targeted mitochondria, leading to increased mitochondrial reactive oxygen species (mROS), decreased mitochondrial membrane potential, and reduced ATP levels. Such exposure resulted in neuronal damage, apoptosis, and neuroinflammation, evidenced by reaction of astrocytes and microglia in the mouse brain (Ma et al., 2024). Similarly, Shin et al. (2023) investigated polystyrene particles (PS-Ps) exposure in adult mice from prenatal development to adult age, using a comprehensive assessment to understand its effects on behavior and to identify potential biological mechanism mediators. The forced swimming test and tail suspension test revealed that the offspring exposed to 10 mg/L PS-Ps spent more immobile time compared to the control group, in line with a depression-like behavior. The study suggests that the exposure to PS-Ps reduces the expression of Gabra2 (an alpha subunit of GABA-A receptors) in the brain of embryonic and adult mice, providing a basis for further exploration of a potential link to behavioral alterations (Shin et al., 2023).

Raeis-Abdollahi et al. (2019) observed increased levels of anxiety and depression-like behaviors after prenatal exposure to chrysotile asbestos in offspring rats and significant structural changes in the hippocampus in male offspring, with reduced cellular proliferation and neuronal maturation, together with dentate gyrus astrogliosis (Raeis-Abdollahi et al., 2019).

Other specific pollutants inducing depression in murine models are Perfluorooctane Sulfonate (PFOS), Tris-(2,3-dibromopropyl) Isocyanurate (TDBP-TAZTO), Melamine Cyanuric Acid (MCA), and NO_2_. An et al. (2023) found that PFOS exposure leads to dose-dependent depressive-like behaviors only in male mice. The study suggests that PFOS may interfere with synaptic plasticity in the hippocampus by affecting the glutamatergic synapse and the CREB/BDNF signaling pathway (An et al., 2023).

Ye et al. (2015) demonstrated neurotoxicity and depression-like behaviors in rats after long-term exposure to Tris-(2,3-dibromopropyl) Isocyanurate (TDBP-TAZTO), linked to an hyperactivation of the HPA axis and increased levels of inflammatory and oxidative stress markers, heightened expression of pro-apoptotic proteins and decreased expression of neurogenesis-related proteins in the hippocampus, damaging the hippocampal neurons in the DG, CA1, and CA3 regions (Ye et al., 2015).

Wang et al. (2021) showed locomotor activity alterations and anxiety- and depression-like behaviors in adolescent male mice after exposure to MCA, probably due to a dysfunction in the hypothalamic-pituitary-thyroid (HPT) axis and modifications in the striatal dopamine function (Wang et al., 2021).

Li et al. (2021) found anxiety and depression-like behaviors in adult mice exposed to NO_2_, with male animals being more affected due to structural and functional abnormalities in myelin sheaths; prolactin might mediate the sex-specific damage of the myelin sheath (Li et al., 2021).

#### 3.2.8 Studies on pesticides and chemicals in non-mammalian animal models

Through an integrated approach combining transcriptomic and metabolomic analyses, Liu et al. (2023) identified the disruption of circadian rhythms, a metabolic imbalance of arginine and proline, and neurotransmitter disorders as the primary biological mechanisms behind the behavioral impairments induced by imidacloprid exposure on adult zebrafish at environmental concentrations (1, 10, and 100 μg/L) (Liu et al., 2023).

Wang et al. (2024) explored the neurotoxic effects of sodium p-perfluorinated nonenoxybenzen sulfonate (OBS) on zebrafish. OBS exposure (32 μg/L) led to poor social behavior, memory decline, and increased cell apoptosis in zebrafish. The pollutant induced mitochondrial dysfunction, decreased mitochondrial membrane potential, and excessive reactive oxygen species production, leading to neuronal damage in the fish brain (Wang et al., 2024).

Focusing on antibiotic medications, Huo et al. (2023) observed changes in gene expression related to neurotransmitter systems (phenylalanine hydroxylase, tyrosine hydroxylase, tryptophan hydroxylase), folate metabolism (spra), and carbonic anhydrase metabolism (ca2, ca4a, ca7, and ca14) as mechanisms linked to the development of depressive-like behaviors in the same animal model after acute exposure to sulfonamides.

Cao et al. (2023) found that exposure to 2,2′,4,4′-Tetrabromodiphenyl Ether (BDE-47), a flame retardant, induces malfunctioning of melanopsin, which in turn affects clock genes, neurotransmitters, hormones, and metabolic enzymes in various brain regions through intrinsically photosensitive retinal ganglion cell (ipRGC) projections, ultimately resulting in depression-like behaviors in the zebrafish larvae (Cao et al., 2023).

Finally, Wu et al. (2024) carried out the only study using reptiles as animal model, by investigating the effect of butyl paraben (BuP) exposure on Chinese striped-neck turtles (Mauremys Sinensis). According to their results, butyl paraben, a chemical commonly used in cosmetics, pharmaceuticals, and food preservation, induced notable behavioral and neurochemical changes in turtle when introduced in their aquatic environment. The investigation highlighted a significant increase in mRNA levels for the serotonin receptor and transporter, as well as tryptophan hydroxylase, suggesting intricate interactions among various neurotransmitter systems. Moreover, the study associated BuP exposure with neuronal death, particularly at elevated concentrations, offering a potential explanation for the observed alterations in behaviour (Wu et al., 2024).

**Fig. 3:**
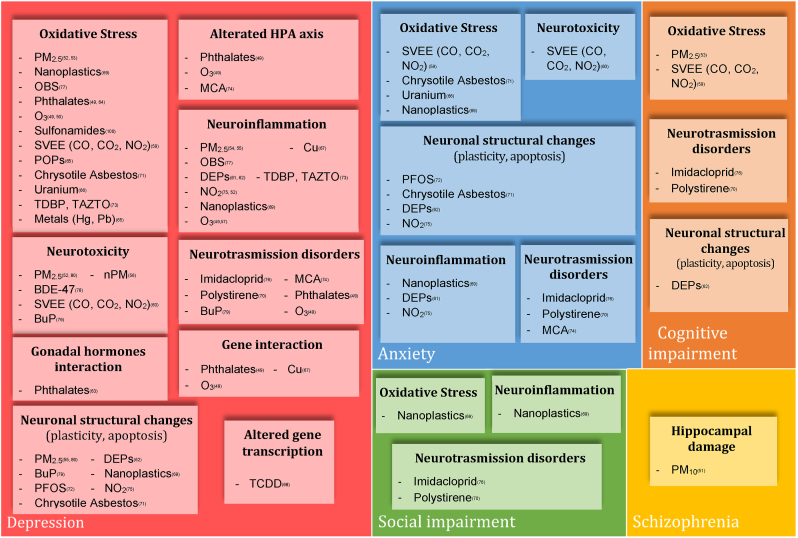
Clinical meaning of pollutants molecular targets-ANIMAL STUDIES.

Fig. 3 summarizes the links between pollutants, mental disorders, and biological mechanisms in humans.

### 3.3 Studies on both human and animal models

Only one paper (Jia et al., 2018) included both human and animal subjects in their study design, establishing the causal relationship between PM exposure and mental disorders through neurotoxicity and mechanisms that involve impaired structural hippocampal changes. The study revealed neurotoxic effects and damage in the neuronal architecture of the hippocampus in mice after PM_2.5_ exposure, highlighting that exposure to significant levels of particulate pollution may exacerbate psychiatric symptoms and increase the likelihood of mood-related behavioral disorders in humans (Jia et al., 2018). Results from this study are included in both Table 1, Table 2

## 4 Discussion

Our review provides an insight on the interaction between exposure to pollutants and mental health. Despite other reviews have already explored the impact of atmospheric pollutants (Borroni et al., 2022b) (Davoudi et al., 2021), to our knowledge, our study is the first focusing on the biological mechanisms linking climatic alterations, environmental pollution, and severe mental disorders by investigating both animal and human models. Our findings highlight that, according to the revised biopsychosocial model of mental health (Engel, 1977) (Tripathi et al., 2019), main mental disorders share underlying biological mechanisms, which operate at various levels, ranging from gene expression alterations and induction of inflammation or oxidative stress to anatomical structural brain changes.

The most consistently studied pollutants are PM_2.5_, PM_10_, O_3_, CO, and CO_2_. Other investigated pollutants, which are present in solid form in the environment, such as phthalates, or contained in water and food, have been less studied. According to our results, the exposure to these pollutants is linked to depressive or psychotic spectrum disorders, increasing inflammatory levels in both human and animal models, mainly through the inflammatory pathways, thus confirming the relationship between inflammation and mental disorders (Harsanyi et al., 2022).

Patients with anxiety and depressive disorders have increased levels of pro-inflammatory cytokines (e.g., TNFα, IL-1α, IL-1β, IL-4, IL-5, IL-6, IL-12, and interferon [IFN]-γ) and C-reactive (Hussain et al., 2016). Cytokines play a role in both the development and maintenance of depressive symptoms, implying that inflammation markers may act as diagnostic or severity indicators in depression (Tamminga, 2023; Voineskos, 2023; Berk, 2023). Evidence shows that inflammatory and metabolic dysregulations can be predictive of a chronic long-term trajectory of depressive disorders in patients on antidepressants (Emsley, 2023) Moreover, chronic low-grade inflammation can reduce the response to pharmacological treatment, thus contributing to worsen the long-term outcome of depressive disorders. In light of this consideration, the impact of pollutants on inflammatory pathways in patients with depression has strong relevance for clinical practice. In fact, our findings highlight that exposure to environmental pollutants, by increasing human inflammatory pathways, could contribute both to worsening symptoms of depression and to significantly reduce the efficacy of pharmacological therapies. Although the number of available studies is limited, this evidence implies that clinicians should consider patients' exposure to environmental pollutants when prescribing medications, considering factors such as geographic residence, dietary habits, and occupational exposures. Such an approach would enable the personalization of therapies in accordance with the principles of precision medicine and personalized therapy.

Inflammation has also been linked to other severe mental illnesses, such as schizophrenia and other psychotic disorders. Xiu et al. (2015) found that higher IL-3 serum levels are associated with more severe symptoms in patients with schizophrenia (Xiu et al., 2015), and Al-Hakeim et al. (2015) found higher levels of inflammatory markers in schizophrenia than in MDD patients and healthy controls. These findings, if confirmed, might suggest the use of anti-inflammatory medications as add-on therapy in psychotic disorders (Zarate, 2023).

The oxidative stress pathway is another biological mechanism linking pollution and mental health (Abi-Dargham et al., 2023). MDD is associated with lower plasma levels of antioxidants, resulting in a higher activity of the pro-inflammatory pathways and apoptotic mediators, such as Caspase-3 and neuronal death. Moreover, it has been reported that antidepressants efficacy could be mediated also by their antioxidative activity (Behr et al., 2012). Thus, it is possible that the use of nutritional supplements containing micronutrients, vitamins, n-3 PUFA and antioxidants may also improve MDD symptoms and prevent their progression.

According to studies included in the review, the dysregulation of the HPA axis could mediate the association between pollutants and mental disorders. Hypersecretion of glucocorticoids (GC) and alteration of the glucocorticoid receptor (GR) have been found in a significant proportion of patients with mental disorders (Cherian et al., 2019). Psychological stress and neuronal activity from the higher cortical regions of the brain are relayed through the limbic system to the hypothalamus, which in turn releases neurotransmitters such as serotonin, norepinephrine and acetylcholine (Yang et al., 2015) (Troubat et al., 2021). This dysregulation confirms a possible link between stress hormones and mental disorders and offers a valuable perspective in new opportunities for prevention and intervention in child and adolescent psychopathology (Danese, 2022; Cortese et al., 2023).

Our research has identified other less frequent mechanisms through which environmental pollutants may lead to the onset of a mental disorder, which include platelet activation, changes in serotonin or dopamine levels, hippocampus damage, and genetic predisposition. In particular, arsenic may triggers depression via platelet activation, while CO exposure depletes serotonin in platelets, leading to depression (Mukherjee et al., 2014; Banerjee et al., 2012). However, these findings come from studies carried out with samples consisting exclusively of women, and exposure to arsenic and CO was in conjunction with PM_2.5_ and PM_10_. Further studies are needed for confirmation, involving mixed-gender populations and**/**or reducing confounding factors such as particulate matter exposure, as applicable. In fact, understanding arsenic and CO co-exposure requires multi-pollutant models to capture real-world interactions. However, advanced statistical approaches, such as multi-level regression or machine learning, could help disentangle the complex relationships between these exposures and health outcomes.

Several studies included in our review have highlighted that several pollutants may influence hippocampal functioning. In particular, studies on animal models have shown that PM_2.5_, DEHP, and PFOS can damage the hippocampus, by altering AR and Erb expression and reducing synaptic plasticity in mice (An et al., 2023; Ehsanifar et al., 2019). Other authors reported alterations in brain biochemistry, particularly in neurotransmitters or genes transcription, as the mechanism linking depression and environmental pollution; for example, pre-exposure to polystyrene particles (PS-P) reduces Gabra2 expression in both embryonic and adult mouse brains (Shin et al., 2023). Similarly, sulfonamides, BDE-47, and imidacloprid disrupt gene expression and neurotransmitter balance in zebrafish (Huo et al., 2023; Cao et al., 2023). However, these findings refer to studies carried out on fishes, a model far from humans, and further research across species is needed.

Some studies have showed that the individual susceptibility to the adverse effects of pollutants may be increased by certain gene polymorphisms; for example, PM_2.5_ has been associated with the re-occurrence of psychotic symptoms in individuals carrying the YWHAB gene polymorphism (locus rs6031849) (Lei et al., 2023); mercury and copper are similarly implicated in neurodevelopmental changes leading to depressive-like behavior in APOE e4 allele carriers (Ng et al., 2015; Xu et al., 2021). Further evidence is needed to show that mental disorders are polygenic diseases with a multifactorial genetic and environmental pathogenesis (Fiorillo and Giordano, 2022).

The novelty of the present review is the fact that we focused on the biological mechanisms linking exposure to environmental pollution and severe mental disorders, by including both animal and human models. This dual approach highlights shared underlying biological mechanisms across species, such as gene expression alterations, inflammation, oxidative stress, and anatomical brain changes. Another strength of our review is the inclusion of studies including large sample sizes, which enhances the reliability of findings related to mental health outcomes. Furthermore, the utilization of biomarkers offers a deeper understanding of the underlying mechanisms, providing a molecular basis for observed clinical phenomena.

This approach facilitates the identification of potential targets for therapeutic intervention, particularly in the context of the "One Health" model, which emphasizes the interconnectedness of human, animal, and environmental health, providing a profound lens through which the biological mechanisms linking environmental pollution to mental disorders can be better understood. Furthermore, it underscores the importance of collaboration among environmental scientists, medical professionals, veterinarians, and other experts to mitigate the impacts of environmental changes on mental health (Yatham, 2023b; Brady and Larrauri, 2023).

The literature reveals progress in understanding these mechanisms, yet gaps persist, particularly regarding the integration of studies on multiple pollutants and the long-term effects of chronic exposure. Future research should focus on standardizing methodologies, expanding studies to diverse populations, and fostering interdisciplinary collaboration to improve generalizability and identify vulnerable groups. Translating these findings into public health policies is crucial for mitigating the impact of environmental stressors on mental health. By addressing these challenges and pursuing innovative strategies, we can better understand and develop interventions to protect mental health in the context of environmental changes.

Our study has some important limitations. First, different tools were used to assess psychiatric disorders, as well as different methods were used for detecting the various biological parameters. In addition, exposure to pollutants was measured by different methods both in human and animal models, making the results poorly reliable and highlighting the need to standardize the studies on exposure to environmental pollutants. It should also be pointed out that - despite the interesting results from animal models - the biological differences between these models and human biology make some findings not applicable to humans. With this regard, the generalizability of findings is limited due to reliance on specific populations or animal models, which may not represent broader human demographics. Caution is needed when extrapolating results across populations or species. Although some studies show similar mechanisms in humans and animals, like the APOE e4 allele's role in neurodevelopment, these findings need further validation across diverse genetic backgrounds and environmental contexts. Furthermore, insufficient data on exposure timing across pregnant dams, young offspring, and adults in many analyzed studies prevented us from organizing results according to developmental timing of pollutant exposure. Finally, all studies carried out on human samples were observational, with a reduced power of evidence.

## 5 Conclusions

Our study highlights key patterns responsible for mental health damage following exposure to polluted environment, serving as a valuable resource for developing new diagnostic and therapeutic strategies. Preventive measures could include the identification of at-risk populations based on geographic areas heavily affected by pollutants or climate change. Additionally, measuring plasma levels of specific environmental toxins in patients with severe mental disorders could help reduce exposure, complementing standard therapies.

While previous reviews have recognized that pollutants and climate change can harm mental health and worsen outcomes for those affected by severe mental disorders (Baker et al., 2024; Radua et al., 2024), our work is pioneering in compiling clinical studies involving both humans and animal models and explaining this correlation through biological disruption. Included studies demonstrate the association between environmental toxins or climate change and provide data on the genetic, biochemical, and anatomical changes responsible for their impact on mental health.

Our study underscores the importance of the “One Health” approach, which advocates for a collaborative, multisectorial, and transdisciplinary approach at local, regional, national, and global levels. By fostering such interdisciplinary partnerships, the 'One Health' approach can play a crucial role in advancing our understanding of and response to the multifaceted challenges posed by environmental disruptions. This methodology aims to achieve optimal health outcomes by acknowledging the interconnection between humans, animals, plants, and their shared environment (CDC About One Health Available).

Our findings reveal that environmental disruptions, such as air and water contamination, destabilize ecosystems and subsequently impact human health, exacerbating severe mental disorders.

## CRediT authorship contribution statement

**Pierluigi Catapano:** Conceptualization, Methodology, Writing – original draft. **Mario Luciano:** Conceptualization, Methodology, Supervision, Writing – review & editing. **Salvatore Cipolla:** Methodology, Writing – original draft. **Daniela D'Amico:** Methodology, Writing – review & editing. **Alessandra Cirino:** Methodology, Writing – original draft. **Maria Chiara Della Corte:** Methodology, Writing – original draft. **Gaia Sampogna:** Methodology, Supervision, Writing – review & editing. **Andrea Fiorillo:** Conceptualization, Supervision, Validation, Visualization, Writing – review & editing.

## Funding sources

This research did not receive any specific grant from funding agencies in the public, commercial, or not-for-profit sectors.

## Declaration of competing interest

The authors declare that they have no known competing financial interests or personal relationships that could have appeared to influence the work reported in this paper.

## Data Availability

Data will be made available upon reasonable request to the corresponding author.
