# Supplementary material for: What is the relationship between exposure to environmental pollutants and severe mental disorders? A systematic review on shared biological pathways
Source: Brain Behav Immun Health. 2024 Dec 12;43:100922. doi: 10.1016/j.bbih.2024.100922 (PMC11719278; doi:10.1016/j.bbih.2024.100922)
Supplement: Multimedia component 1 [file mmc1.docx]

| Table S1. Risk of bias assessments in observational studies | | | | | | | | |
| --- | --- | --- | --- | --- | --- | --- | --- | --- |
| **Author  (year)**  *type of study*  ***(Country)*** | **Risk of Bias assessed using ROBINS-E tool** | | | | | | | |
|  | ***Bias due to confounding*** | ***Bias arising from measurement of the exposure*** | ***Bias in selection of participants*** | ***Bias due to post-exposure interventions*** | ***Bias due to missing data*** | ***Bias arising from measurement of the outcome*** | ***Bias in selection of the reported result*** | **Overall  Risk of Bias** |
| **Xu**  **(2024)**  *Cohort study*  ***(China)*** | Low | Some concerns | Low | Low | Low | Low | Low | Low risk of bias except for some concerns about the measurement of the exposure |
| **Abo-el-Ata**  **(2023)**  *Cross-sectional study*  ***(Egypt)*** | Low | Some concerns | Some concerns | Low | Some concerns | Low | Low | Some concerns |
| **Bjørklund**  **(2023)**  *Cross-sectional study*  ***(Kazakhstan)*** | Low | High | Low | Low | Low | Low | Low | High |
| **Lei**  **(2023)**  *Historical cohort study*  ***(China)*** | Some concerns | High | Some concerns | Low | Low | Some concerns | Low | High |
| **Yuan**  **(2023)**  *Observational prospective study*  ***(China)*** | Some concerns | Some concerns | Some concerns | Low | Low | Low | Low | Some concerns |
| **Bao**  **(2022)**  *Cohort study*  ***(China)*** | Some concerns | Some concerns | Some concerns | Low | Some concerns | Some concerns | Some concerns | Some concerns |
| **Ahlers**  **(2021)**  *Cohort study*  ***(USA)*** | Some concerns | Some concerns | Some concerns | Low | Low | Low | Low | Some concerns |
| **Dridi**  **(2020)**  *Prospective cohort study*  ***(France)*** | Some concerns | Some concerns | Low | Low | Low | Low | Low | Some concerns |
| **Gao**  **(2020)**  *Prospective cohort study*  ***(China)*** | Low | Low | Low | Low | Low | Low | Low | Low |
| **Salvi**  **(2020)**  *Cohort study*  ***(USA)*** | Some concerns | Low | Low | Low | Low | Low | Low | Low risk of bias except for some concerns due to confounding |
| **Chu**  **(2019)**  *Prospective cohort study*  ***(China)*** | Some concerns | Low | Low | Low | Low | Low | Low | Low risk of bias except for some concerns due to confounding |
| **Ehsanifar**  **(2019)**  *Longitudinal cohort study*  ***(Iran)*** | Some concerns | Low | Low | Low | Low | Low | Low | Low risk of bias except for some concerns due to confounding |
| **Gaum**  **(2019)**  *Observational longitudinal study* ***(Germany)*** | Low | Low | Low | Low | Low | Low | Low | Low |
| **Gaum**  **(2017)**  *Observational longitudinal study*  ***(Germany)*** | Low | Low | Low | Low | Low | Low | Low | Low |
| **Lestaevel**  **(2015)**  *Case-control study*  ***(France)*** | Low | Low | Low | Low | Low | Low | Low | Low |
| **Ng**  **(2015)**  *Prospective cohort study*  ***(Taiwan)*** | Some concerns | Low | Low | Low | Some concerns | Low | Low | Some concerns |
| **Ye**  **(2015)**  *Case-control study*  ***(China)*** | Low | Low | Low | Low | Low | Low | Low | Low |
| **Mukherjee**  **(2014)**  *Cross-sectional study*  ***(India)*** | Some concerns | Some concerns | Low | Low | Low | Low | Low | Some concerns |
| **Banerjee**  **(2012)**  *Cross-sectional study*  ***(India)*** | Low | Low | Low | Low | Low | Low | Low | Low |
| **Tomei**  **(2007)**  *Case-control study*  ***(Italy)*** | Some concerns | Some concerns | Low | Low | Some concerns | Some concerns | Low | Some concerns |

| Table S2. Risk of bias assessment in experimental studies (NRSI) | | | | | | |
| --- | --- | --- | --- | --- | --- | --- |
| **Author**  **(year)**  *type of study*  ***(Country)*** | **Risk of Bias assessed using RoB II Tool** | | | | | |
|  | ***Bias arising from the randomization process*** | ***Bias due to deviations from intended interventions*** | ***Bias due to missing outcome data*** | ***Bias in measurement of the outcome*** | ***Bias in selection of the reported result*** | **Overall Risk of Bias** |
| **Choi**  **(2024)**  *Experimental study*  ***(China)*** | Moderate | Low | Low | Low | Low | Moderate |
| **Debler**  **(2024)**  *Experimental study*  ***(China)*** | Low | Low | Low | Moderate | Low | Moderate |
| **Ma**  **(2024)**  *Experimental study*  ***(China)*** | Low | Moderate | Low | Low | Low | Moderate |
| **Wang**  **(2024)**  *Experimental study*  ***(China)*** | Low | Low | Low | Moderate | Low | Moderate |
| **Wu**  **(2024)**  *Experimental study*  ***(China)*** | Low | Low | Moderate | Low | Low | Moderate |

| **Xie**  **(2024)**  *Experimental study*  ***(China)*** | Moderate | Low | Low | Moderate | Low | Moderate |
| --- | --- | --- | --- | --- | --- | --- |
| **An**  **(2023)**  *Experimental study*  ***(China)*** | Low | Low | Low | Moderate | Low | Moderate |
| **Baumann**  **(2023)**  *Experimental study*  ***(USA)*** | Low | Moderate | Low | Low | Low | Moderate |
| **Cao**  **(2023)**  *Experimental study*  ***(China)*** | Low | Moderate | Low | Low | Low | Moderate |
| **Huo**  **(2023)**  *Experimental study*  ***(China)*** | Moderate | Low | Low | Low | Low | Moderate |
| **Liu**  **(2023)**  *Experimental study*  ***(China)*** | Low | Low | Low | Low | Moderate | Moderate |
| **Shin**  **(2023)**  *Experimental study*  ***(Korea)*** | Low | Moderate | Low | Low | Low | Moderate |
| **Ehsanifar**  **(2022)**  *Experimental study*  ***(Iran)*** | Low | Moderate | Low | Low | Low | Moderate |
| **Ji**  **(2022)**  *Experimental study*  ***(China)*** | Low | Moderate | Low | Low | Low | Moderate |
| **Wei**  **(2022)**  Clinical trial  **(China)** | Moderate | Moderate | Low | Low | Low | Moderate |
| **Kochi**  **(2021)**  *Experimental study*  ***(USA)*** | Moderate | Low | Low | Low | Low | Moderate |
| **Li**  **(2021)**  *Experimental study*  ***(China)*** | Low | Moderate | Low | Low | Low | Moderate |
| **Wang**  **(2021)**  *Experimental study*  ***(China)*** | Moderate | Moderate | Low | Low | Low | Moderate |
| **Xu**  **(2021)**  *Experimental study*  ***(China)*** | Low | Moderate | Low | Low | Low | Moderate |
| **Raeis-Abdollahi (2019)**  *Experimental study*  ***(Iran)*** | Low | Moderate | Low | Low | Low | Moderate |
| **Jia**  **(2018)**  *Experimental study*  **(China)** | Moderate | Low | Low | Low | Low | Moderate |
| **Liu**  **(2018)**  *Experimental study* ***(China)*** | Low | Moderate | Low | Low | Low | Moderate |
| **Xu**  **(2015)**  *Experimental study*  ***(China)*** | Moderate | Moderate | Low | Low | Low | Moderate |
| **Zuo**  **(2014)**  *Experimental study*  ***(China)*** | Low | Moderate | Low | Low | Low | Moderate |
| **Davis**  **(2013)**  *Experimental study* ***(USA)*** | Low | Moderate | Low | Low | Low | Moderate |
| **Fonken**  **(2011)**  *Experimental study* ***(USA)*** | Moderate | Moderate | Low | Low | Low | Moderate |
| **Mokoena**  **(2010)**  *Experimental study*  ***(South Africa)*** | Moderate | Moderate | Low | Moderate | Low | Moderate |

**= Xu, X.; Yang, Y.; Wang, R.; Wang, Y.; Ruan, Q.; Lu, Y. Perinatal Exposure to Di-(2-Ethylhexyl) Phthalate Affects Anxiety- and Depression-like Behaviors in Mice. *Chemosphere* **2015**, *124*, 22–31, doi:10.1016/j.chemosphere.2014.10.056
